# Intestinal Barrier Dysfunction Develops at the Onset of Experimental Autoimmune Encephalomyelitis, and Can Be Induced by Adoptive Transfer of Auto-Reactive T Cells

**DOI:** 10.1371/journal.pone.0106335

**Published:** 2014-09-03

**Authors:** Mehrnaz Nouri, Anders Bredberg, Björn Weström, Shahram Lavasani

**Affiliations:** 1 Department of Clinical Sciences, Clinical Research Centre, Surgery Research Unit, Lund University, Malmö, Sweden; 2 Department of Biology, Lund University, Lund, Sweden; 3 ImmuneBiotech AB, Lund Life Science Incubator, Medicon Village, Lund, Sweden; 4 Department of Laboratory Medicine, Section of Medical Microbiology, Lund University, Malmö, Sweden; Uniform Services University of the Health Sciences, United States of America

## Abstract

Multiple sclerosis (MS) is a chronic inflammatory demyelinating disease of the central nervous system with a pathogenesis involving a dysfunctional blood-brain barrier and myelin-specific, autoreactive T cells. Although the commensal microbiota seems to affect its pathogenesis, regulation of the interactions between luminal antigens and mucosal immune elements remains unclear. Herein, we investigated whether the intestinal mucosal barrier is also targeted in this disease. Experimental autoimmune encephalomyelitis (EAE), the prototypic animal model of MS, was induced either by active immunization or by adoptive transfer of autoreactive T cells isolated from these mice. We show increased intestinal permeability, overexpression of the tight junction protein zonulin and alterations in intestinal morphology (increased crypt depth and thickness of the submucosa and muscularis layers). These intestinal manifestations were seen at 7 days (i.e., preceding the onset of neurological symptoms) and at 14 days (i.e., at the stage of paralysis) after immunization. We also demonstrate an increased infiltration of proinflammatory Th1/Th17 cells and a reduced regulatory T cell number in the gut lamina propria, Peyer's patches and mesenteric lymph nodes. Adoptive transfer to healthy mice of encephalitogenic T cells, isolated from EAE-diseased animals, led to intestinal changes similar to those resulting from the immunization procedure. Our findings show that disruption of intestinal homeostasis is an early and immune-mediated event in EAE. We propose that this intestinal dysfunction may act to support disease progression, and thus represent a potential therapeutic target in MS. In particular, an increased understanding of the regulation of tight junctions at the blood-brain barrier and in the intestinal wall may be crucial for design of future innovative therapies.

## Introduction

There is growing evidence for a paradigm shift in our view on the pathogenesis of autoimmune diseases. In addition to genetic susceptibility, making the individual react abnormally to self antigens, the loss of the protective function of epithelial barriers that interact with the environment, not least the gastrointestinal mucosa, seems to be involved in the development of autoimmunity [1]. Recent observations in humans and in a variety of animal models indicate that an increased intestinal permeability (IP), often referred to as a “leaky gut”, is playing a pathogenic role not only in development of gastrointestinal disorders like inflammatory bowel disease (IBD) and celiac disease, but also in systemic autoimmune diseases, like type 1 diabetes (T1D) [1], [2], [3], [4].

Multiple sclerosis (MS) is one of the inflammatory autoimmune disorders with an increasing incidence. MS is characterized by breakdown of the blood-brain barrier (BBB) and demyelination of the central nervous system (CNS) due to infiltrating self-reactive T cells recognizing myelin antigens. The etiology of MS is unknown, however, epidemiological and genetic studies suggest that MS is provoked following exposure to environmental factors, which are potentially responsible for loss of tolerance and peripheral activation of myelin-specific T cells [5], [6]. Genome-wide association studies (GWAS) have confirmed the complexity of MS and uncovered immune-related gene variants linked also to other autoimmune diseases, such as T1D and IBD [7]. The association between MS and IBD is strengthened by observations of an increased incidence of IBD, including both Crohn's disease (CD) and ulcerative colitis (UC), among MS patients [8], [9]. The effect of antibiotic treatment on the severity of an experimental colitis model for IBD, and on the experimental autoimmune encephalomyelitis (EAE) animal model of MS employed in the present work, indicates a strong influence of the gut and the commensal bacteria on the immune system, suggesting that disturbances in gut physiology may contribute to development of these diseases [10], [11].

IBD is characterized by a chronic inflammation of the gastrointestinal tract and alterations of IP [3]. The role of loss of intestinal barrier function has not been established, but increased IP seems to cause an abnormality in antigen delivery that may in turn trigger a multi-organ process leading to the autoimmune responses. The macromolecular passage over the intestinal epithelium may follow transcellular and/or paracellular routes, the former by vesicular transport - transcytosis, and the latter via the tight junctions (TJ) between the epithelial cells [12]. The precise regulation of TJ is not completely understood but the protein zonulin has been shown to regulate intracellular signaling leading to rapid and reversible opening of the intestinal TJ [4], [13]. Several human and experimental autoimmune animal models, such as celiac disease and T1D have been characterized by TJ dysfunction and elevated levels of zonulin expression [4], [14]. Inflammatory cytokines, such as IFN-γ and TNF-α, have been shown to increase permeability across the endothelial and epithelial layers and to have a regulatory effect on zonulin [15], [16].

EAE induced with myelin oligodendrocyte glycoprotein (MOG) is a model for MS in rodents with clinical and pathological features closely similar to the human disease [17]. EAE has been a valuable model in investigating the pathogenesis and searching for new therapies [17]. Development of EAE has been thought to require IFN-γ producing Th1 cells, however, Th17 cells have recently been recognized as an essential subpopulation in EAE as well as in MS, T1D and IBD [18]. IL-17A production in CNS-infiltrating T cells has been associated with BBB disruption and inflammatory CD4^+^ T cell recruitment into the CNS [19]. The intestinal inflammation characteristic of IBD and the immunopathological effects of Th17 cells have been explained by overproduction of proinflammatory cytokines, such as TNF-α and IL-6 being released mainly by macrophages, though IL-6 acting together with TGF-β mediates the differentiation of Th17 cells [20].

Focusing on the mucosal immune system, we have recently reported a potential therapeutic strategy for MS by oral administration of a mixture of probiotic *Lactobacillus* species which suppressed established EAE disease [21]. The treatment resulted in IL-10-dependent activation of regulatory T cells (Tregs) in gut-related lymphoid organs as well as the CNS, followed by reductions in numbers of inflammatory cells and levels of IFN-γ, TNF-α and IL-17. Our previous results thus showed that treatment targeting the gut of EAE mice can mediate a systemic health effect suppressing the chronic inflammation.

In the present study we tested a hypothesis that EAE is accompanied by a disrupted mucosal immune homeostasis. We report an increased IP preceding EAE onset and further escalating during the progression of the disease. Our subsequent analysis of the small intestine demonstrated morphological changes and altered expression of the TJ modulator protein zonulin. We also found a proinflammatory disruption of the mucosal balance between Th1/Th17 and Treg cell populations in intestinal lamina propria, Peyer's patches and mesenteric lymph nodes (MLN). Importantly, we also demonstrate that adoptive transfer of encephalitogenic T cells induces all these EAE signs of intestinal barrier damage, no less severe than those seen with active immunization.

## Results

### Increased intestinal permeability

**Figure 1:**
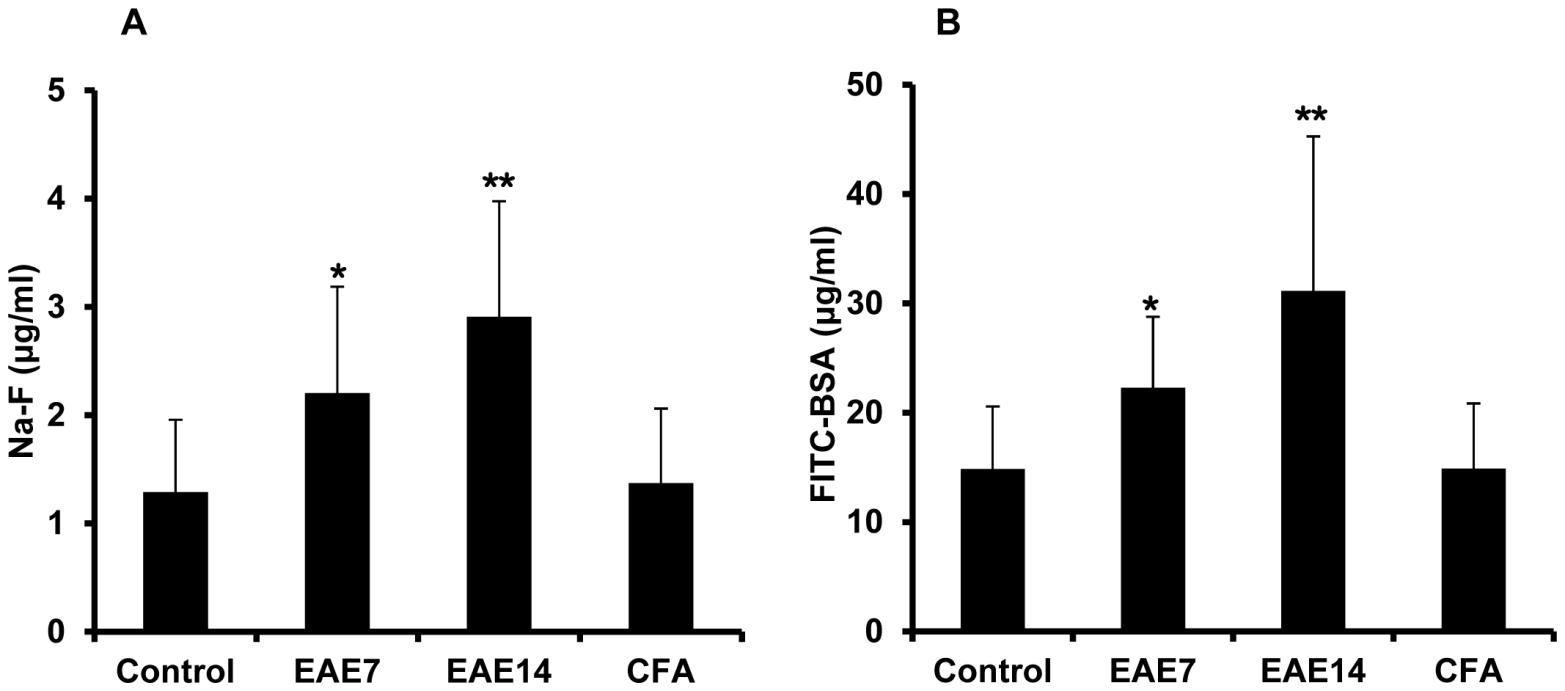
Increased plasma levels of intestinal permeability markers during EAE progression. Sodium fluorescein, Na-F (MW 376 Da) (A), FITC-BSA (MW 66 KDa) (B) in unimmunized animals (control), EAE mice at day 7 (EAE7) and day 14 (EAE14) following immunization, and mice at day 14 after immunization with CFA without MOG followed by administration of pertussis toxin (CFA). Marker levels were measured in plasma samples taken at 1 h after gavage with a saline solution containing the indicated marker for Na-F and 2 h for FITC-BSA. The results are expressed as mean ±SD, (n = 7–10). * represents a p-value≤0.05, ** a p-value≤0.01.

When EAE is actively induced by immunizing C57BL/6 mice, a disease incidence of approximately 80% is expected and the animals usually lose around 10% of their body weight, preceding by a few days the neurological symptoms which appear at 8–10 days after immunization. On this basis, the animals were weighed and scored for clinical signs of disease daily and only those with weight loss (amounting to about 10%) were included at day 7. By day 14, most animals had developed paralysis. IP was assessed *in vivo* in mice shortly before clinical onset, at day 7 post immunization (EAE7), and after establishment of disease, at day 14 (EAE14) (Fig. 1 A-B). The marker molecules Na-F and FITC-BSA were gavaged to these groups of animals and the concentration of the markers was measured in blood samples. In comparison to unimmunized healthy controls, plasma levels of Na-F (Fig. 1 A) and FITC-BSA (Fig. 1 B) were both significantly increased in the EAE7 group and were further enhanced among the EAE14 mice. These marker molecules were not affected in another set of control animals following an identical immunization protocol using CFA and pertussis toxin administration, but without the MOG peptide (CFA). The data indicate increased IP to both the small molecule and the macromolecular protein marker already before the clinical onset of EAE, that became enhanced during the progression of the disease.

### Altered intestinal morphology

**Figure 2:**
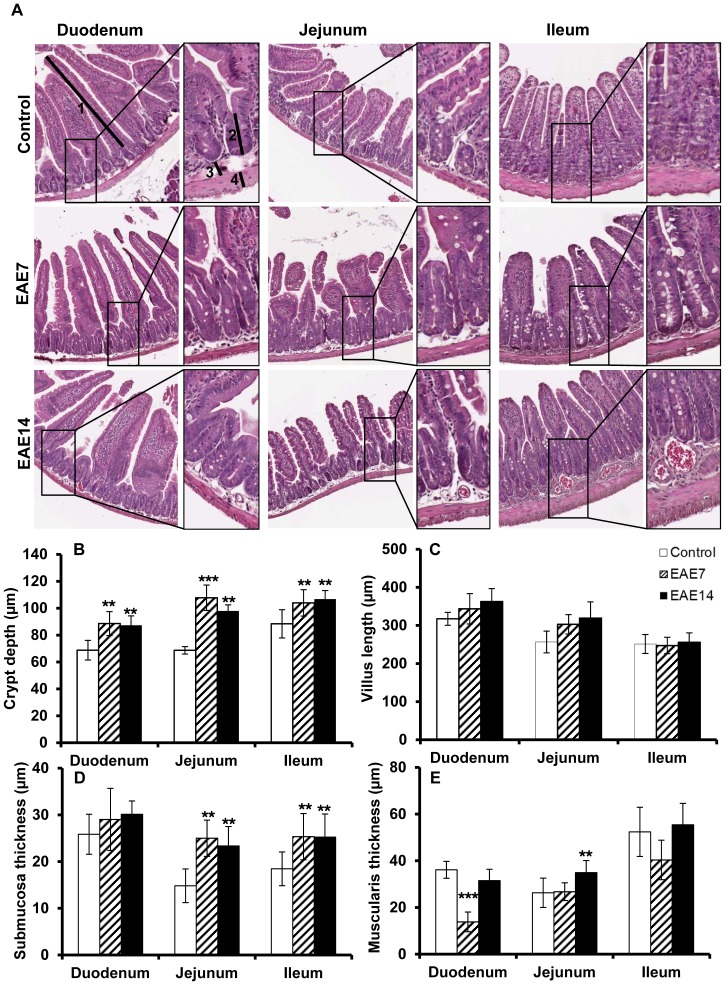
Altered intestinal architecture in the small intestine. H&E-stained sections from duodenum, jejunum and ileum were isolated from control, EAE7 and EAE14 animals (A). The sections were examined for crypt depth, defined as the length from crypt base to villus-crypt junction (B), villi length (C), submucosa (D) and muscularis thickness (E). Arrows demonstrate approximate measurements for villus length (1) crypt depth (2) submucosa thickness (3) and muscularis thickness (4) (A, original magnification ×40, insets ×100). Each bar represents mean ±SD of 7–9 analyzed sections per animal, (n = 5). **represents a p-value≤0.01 and *** a p-value≤0.001.

Our observation of increased IP indicates alterations of intestinal barrier properties and we therefore studied intestinal morphology during the disease progression. Sections from the duodenum, jejunum and ileum revealed significantly increased crypt depths all over the small intestine in animals with EAE compared to control mice (Fig. 2 A and B), while the villus length in duodenum and jejunum of EAE mice was slightly increased (not significantly) (Fig. 2 A and C). Furthermore, the submucosal layer in jejunum and ileum of diseased animals was significantly enlarged (Fig. 2 A and D). On the other hand, analysis of muscularis revealed a decreased thickness in EAE7 (significant values only in duodenum) to become increased (significant values only in jejunum) in EAE14 (Fig. 2 E). Taken together, these data show a correlation between EAE and altered intestinal morphology and barrier function. The changes were evident already before the onset and remained after the establishment of the disease.

### Intestinal overexpression of tight junction regulator zonulin

**Figure 3:**
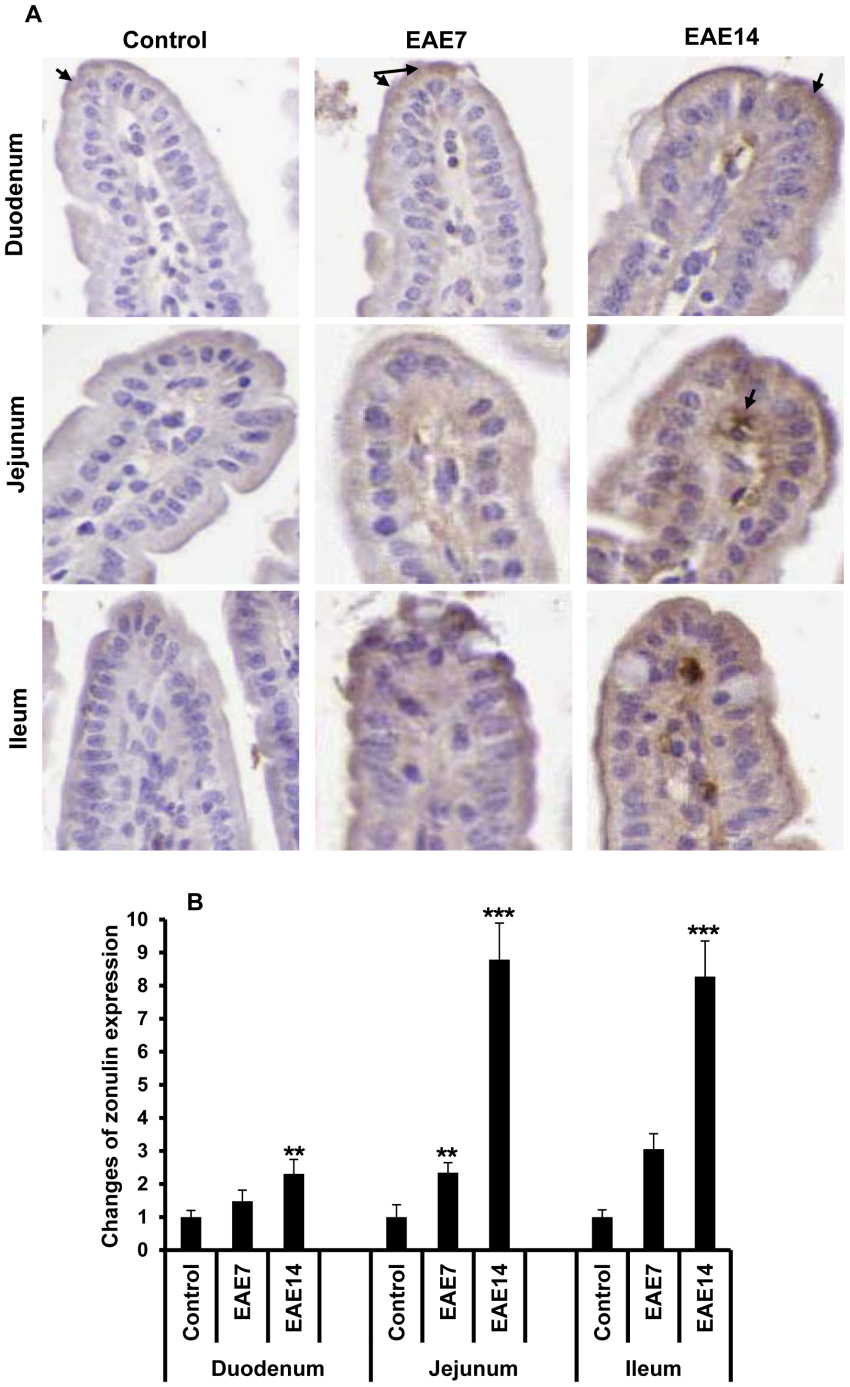
Increased zonulin expression in the small intestine. Immunohistochemical staining of zonulin in sections from duodenum, jejunum and ileum, from healthy controls, EAE7 and EAE14 mice (A). Arrows show zonulin both in enterocytes and lamina propria on top of the villi. Semi-quantitative analysis of zonulin staining (B). Staining intensity was expressed as positive pixels/mm^2^ and converted as ratio to the mean values from relevant sections in the healthy control animals. Data shown are mean ±SD from 4–6 animals for each group. ** represents a p-value≤0.01 and *** a p-value≤0.001 between control and EAE7 or EAE14 animals.

Several recent reports have shown a major role of intercellular TJ in regulation of IP. To gain further understanding of the intestinal functional and morphological changes during establishment of EAE, we investigated the expression of zonulin by immunohistochemical staining (Fig. 3). An increased expression of zonulin in the duodenum, jejunum and ileum of EAE mice was demonstrated, compared to the controls. The overexpression of zonulin was confirmed in both intestinal epithelial cells and lamina propria cells; and with a similar staining pattern as previously demonstrated in patients with celiac disease [15]. Semi-quantitative analysis revealed that the zonulin expression was increased prior to onset of EAE and then augmented, mostly in the jejunal and ileal parts of the intestine, after establishment of the disease. The results show that increased zonulin expression in the small intestine coincides with the morphology and IP alterations found by us to precede the onset of EAE.

### Disturbed intestinal T cell homeostasis

**Figure 4:**
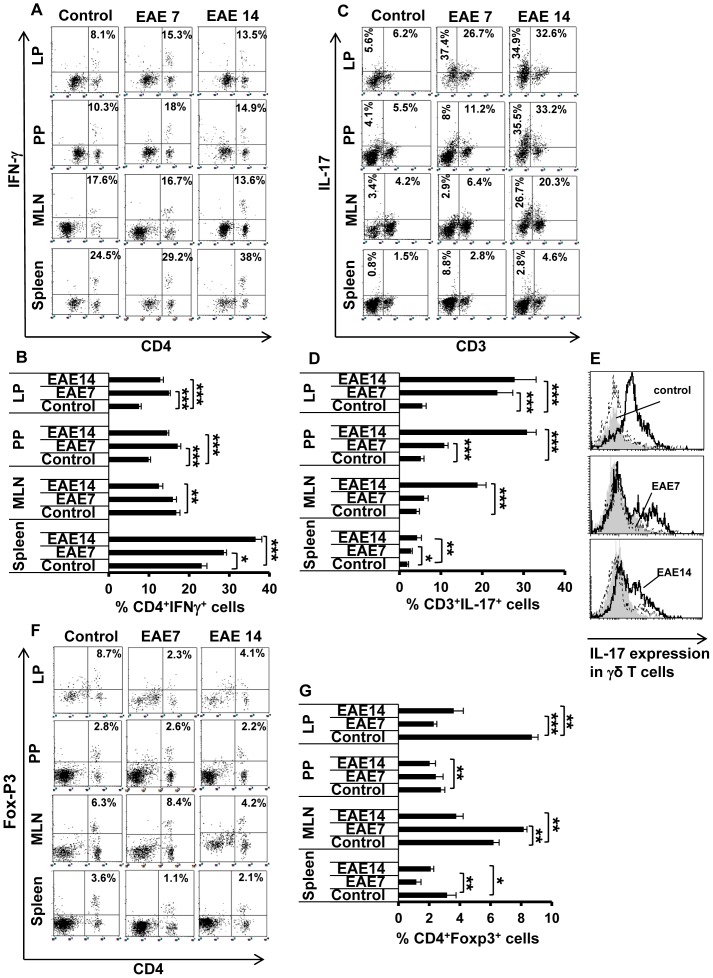
Increased peripheral and intestinal inflammation. Intestinal lamina propria (LP), Peyer's patches (PP), mesenteric lymph nodes (MLN) and spleen were isolated from unimmunized controls and EAE7 and EAE14 mice. Single cell suspensions were prepared and analyzed by flow cytometry for presence of T cells expressing IFN-γ, IL-17, or Foxp3. The lymphocytes were gated and cells were analyzed for expression of the indicated cytokines. The dot plots (A, C, and F) show the percentage of double positive cells (upper right quadrants) out of total T cells and the data are representative of one of three independent experiments. The histograms (B, D, and G) show results from all performed experiments, mean ±SD (n = 5). The frequency of CD4^+^IFN-γ^+^T cells (A) and relative percentage (B). The frequency of CD3^+^IL-17^+^ T cells (C; upper right), CD3^−^IL-17^+^ cells (C; upper left) and relative percentage of CD3^+^IL-17^+^ T cells (D). A histogram shows IL-17 expression by gated γδ T cells (E). The frequency of CD4^+^ Foxp3^+^ Tregs, dot plots from one representative experiment (F) and total results (G). * represents a p-value≤0.05, ** a p-value≤0.01 and *** a p-value≤0.001 in comparison with the controls.

Increased IP and alterations in intestinal TJ have previously been associated with immunological activities in the small intestine and related lymphoid organs [15]. We performed flow cytometry and found increased levels of CD4^+^ IFN-γ^+^ (Th1) cells (almost two-fold) in the LP, PP and the spleen during the development of EAE (days 7 and 14) compared to control healthy animals, while there was no change in MLN. Also the level of the IL-17 expressing CD3^+^ T cells was increased at all these lymphoid sites (Fig. 4 C and D), already before the onset of the disease (EAE7), with highest frequency in LP (more than four-fold elevation) and lowest in the spleen (less than two-fold). This effect was enhanced in the EAE14 group. A major source of IL-17 producing T cells is Th17 cells but intestinal γδ T cells are also releasing IL-17. Accordingly, we found increased expression of IL-17 in γδ T cells, mainly in LP but also in PP and MLN, and only during the EAE14 phase of the disease (Fig. 4 E). In line with this finding, we noticed strong expansion of non-T cell-sources of IL-17 (CD3^−^IL-17^+^ cells), as inferred from the data on CD3^−^ cells presented in Figure 4 C; starting at the early phase (EAE7) in LP and further increasing in PP and MLN at the EAE14 stage (approximately seven-fold, as exemplified by the increase from 3.4% to 26.7% in MLN). These results suggest that EAE is associated with induction not only of peripheral IL-17-producing cells but the activation of these cells in the intestine.

Recent studies have suggested that Tregs retain developmental plasticity with a potential to lose the Foxp3 expression and reprogram into Th17 effector cells particularly in the gut microenvironment [22]. Consistent with our findings of expansion of Th17 cells we therefore analyzed the frequency of Foxp3^+^CD4^+^ Tregs. The results revealed decreased levels of Tregs in all tissues (Fig. 4 F and G), most prominent in LP as well as in spleen already before the onset (EAE7). The Treg levels slightly recovered during the late phase of the disease (EAE14) but still remained lower than in control animals. Overall, our data demonstrate a marked increase of IL17 producing cells and a more modest Th1 activity concomitant with reduced levels of Foxp3 expressing Tregs in the intestine and related lymphoid tissues during development of EAE.

### Increased expression of IL-6 and TNF-α in mucosal macrophages and dendritic cells

Recent work has demonstrated that Th1-derived IFN-γ can trigger antigen-presenting cells (APC) to produce cytokines which favor Th17 cell differentiation. In addition, IL-6 has been shown to play a key role in the development or maintenance of Th17 cells in the intestine which in concert with TGF-β may suppress Th1 and promote reprogramming of Tregs into Th17 [23]. Moreover, TNF-α can further drive Th-17 generation in the presence of IL-6 and TGF-β [24]. IL-6 and TNF-α are produced by macrophages and dendritic cells (DC).

**Figure 5:**
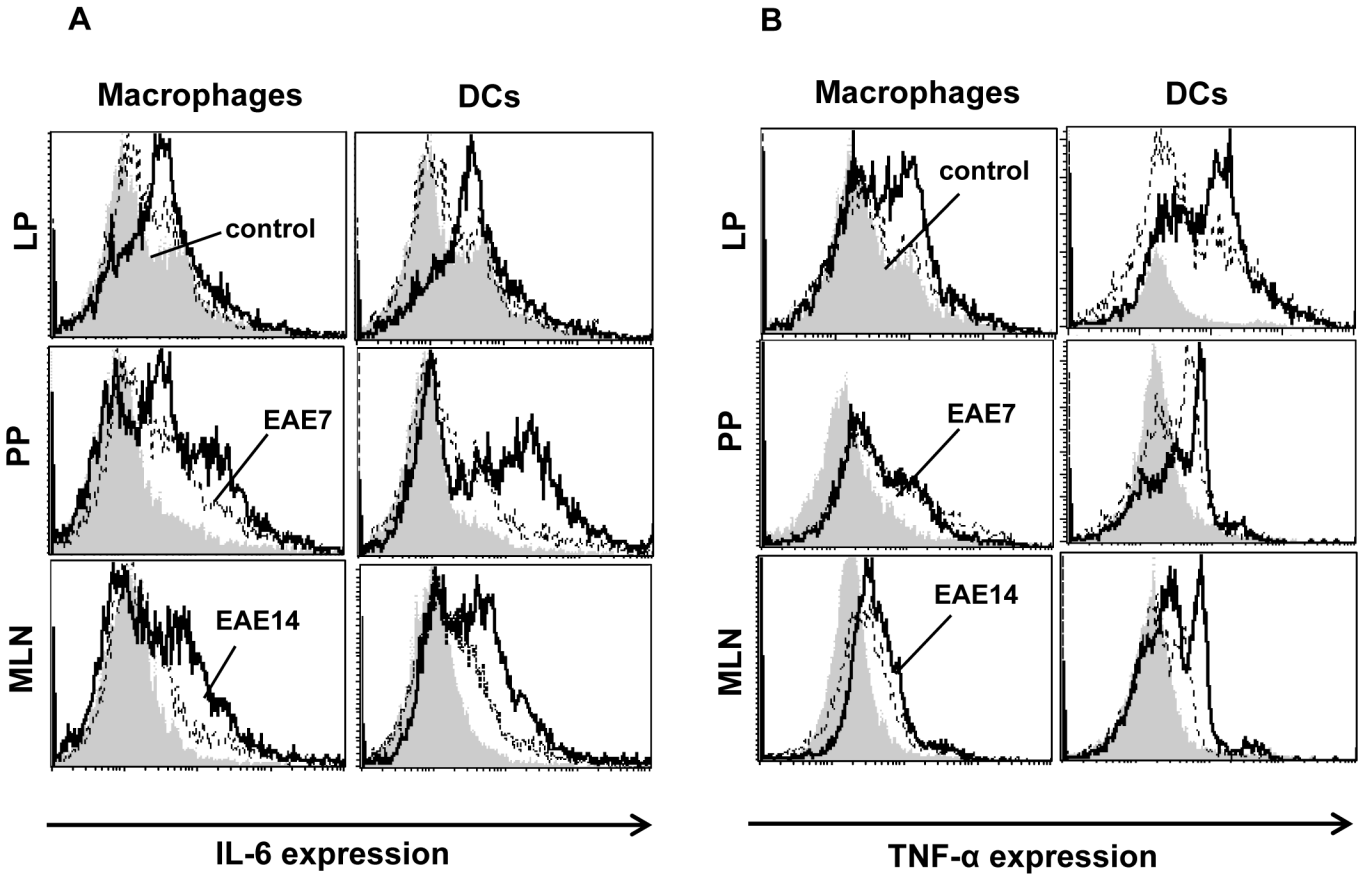
Increased IL-6 and TNF-α expression in intestinal macrophages and dendritic cells. Intestinal lamina propria (LP), Peyer's patches (PP) and mesenteric lymph nodes (MLN) isolated from unimmunized controls, and EAE7 and EAE14 mice were analyzed by flow cytometry for presence of F4/80^+^ macrophages and CD11c^+^ DC expressing IL-6 (A) and TNF-α (B). Data are representative of one of three independent experiments.

We examined the cytokine profile of the F4/80 expressing macrophages and CD11c^+^ DC in LP, PP and MLN of the mice at the indicated time points after immunization (Fig. 5 A and B). There was a minor upregulation of IL-6 in macrophages and DC in all organs at day 7 (EAE7) which markedly increased after the progression of the disease (Fig. 5 A). IL-6 upregulation was most prominent in APC from PP and MLN. Consistent with the increased IL-6 levels, the TNF-α expression in macrophages and DC was also enhanced in all organs (Fig. 5 B), although most prominent in DC and those from LP in particular.

Taken together, the data reveal evidence of activated APC in the LP and gut associated lymphoid tissues (GALT), releasing IL-6 and TNF-α which create a cytokine milieu that can favor the generation of inflammatory IL-17 producing cells.

### Disease specific T cells cause loss of intestinal barrier function

**Figure 6:**
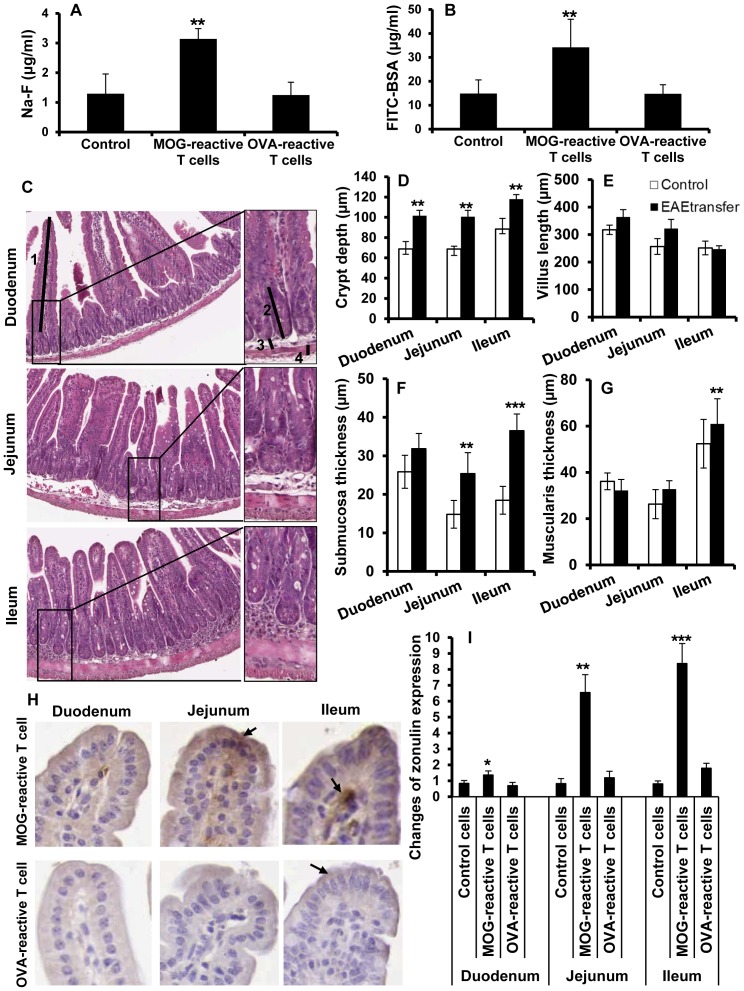
Increased intestinal permeability and altered intestinal morphology after adoptive transfer of encephalitogenic T cells. Na-F (A) , FITC-BSA (B) in plasma from mice receiving un-stimulated lymph node cells (Control), MOG-reactive T cells (adoptively transferred EAE) and OVA-reactive T cells, (n = 3–5). Mice were gavaged with a marker molecules as described in Figure 1. H&E-sections from duodenum, jejunum and ileum isolated from animals receiving MOG-reactive T cells (EAETransfer) (C). The sections were examined for crypt depth (D), villus length (E), submucosa (F) and muscularis thickness (G). Arrows demonstrate approximate measurements for villus length (1) crypt depth (2) submucosa thickness (3), muscularis thickness (4) and highlight the differences between the groups (C, original magnification ×40, insets ×100). Each bar represents mean ±SD of 7–9 analyzed sections per animal, (n = 5). Immunohistochemical analysis of zonulin expression from mice receiving MOG-reactive and OVA-reactive T cells (H). Arrows show zonulin both in enterocytes and lamina propria on top of the villi. Semi-quantitative analysis of zonulin staining (I). Staining intensity was expressed as positive pixels/mm^2^ and converted as ratio to the mean values from relevant sections in the control animals. Data shown are mean ±SD from 3–5 animals for each group. ** represents a p-value≤0.01 and *** a p-value≤0.001.

Development of EAE is associated with uncontrolled T cell expansion. To further study the role of autoreactive encephalitogenic T cells on intestinal barrier function and TJ regulation we induced EAE by adoptive transfer of such encephalitogenic CD4^+^ T cells that had been stimulated with MOG_35–55_ in presence of IL-12 and IL-2, while control animals received freshly prepared lymphoid cells from naive animals. At day 14 post transfer, animals were investigated for IP changes by gavage of marker molecules as mentioned above. Significantly elevated plasma levels of both Na-F (Fig. 6 A) and FITC-BSA (Fig. 6 B) were found in diseased animals, similar to the levels in animals with actively induced disease at day 14 post immunization. To determine the necessity for autoreactivity of the T cells in disruption of intestinal homeostasis we also examined the relative capacity of T cells specific for a non-self antigen. We found that OVA-reactive T cells adoptively transferred to naive animals were incapable of increasing IP (Fig. 6 A and B). Histological examination of H&E stained intestinal sections revealed noticeable morphological alterations in mice with adoptively transferred EAE (EAEtransfer) (Fig. 6 C). Progression of EAE in these mice resulted in significantly increased crypt depths overall in the small intestine (Fig. 6 D) and increased villus length in duodenum and jejunum (Fig. 6 E). Further analysis also revealed significantly enlarged submucosal thickness in jejunum and ileum (Fig. 6 F) and enlarged muscularis mainly in ileum (Fig. 6 G). These changes were similar to those in EAE mice at day 14 after active immunization. Interestingly, an increased expression of zonulin was also demonstrated in intestinal specimens including duodenum, jejunum and ileum in mice with adoptively transferred MOG-reactive T cells, compared to those receiving OVA-reactive cells (Fig. 6 H). Semi-quantitative analysis revealed that the zonulin overexpression in mice with adoptively transferred EAE was as strong as in samples from actively immunized animals at day 14 post immunization. These findings show that circulating autoreactive T cells play an essential role for the pathological changes in intestinal wall morphology and barrier properties.

## Discussion

In the present study we examined the intestinal tract of mice with EAE, the prototype animal model for MS, and demonstrate an increased gut permeability and an altered mucosal structure including inflammation in the small intestine. Our result correlates well with a non-reproduced study from 1996 suggesting an increased IP in 5 of 20 studied MS patients [25], as well as with observations from CD and T1D patients where an increased IP precedes clinical onset and relapses [15]. Similar findings have also been demonstrated in animal models for IBD and T1D [14], [15]. We therefore examined the histology of the small intestine from EAE animals. The data revealed a tendency for sequential histological alterations starting early during the disease and coinciding with IP changes; and the jejunum and ileum seem to be the more affected segments. Possibly, the decrease of the thickness of the muscularis in the duodenum and the ileum, observed already early during the disease but to display regress at a later phase, could be due to muscularis dystrophy at onset and immune cell infiltration in the submucosal region during progression. The mechanisms behind the EAE-induced derangement of the normal villus-crypt structure is not clear but could be related to differentiation of the intestinal subepithelial myofibroblasts, cell-volume changes in the enterocytes, or alteration of crypt stem cell proliferation.

The recently identified modulator of IP named zonulin represents a human homologue to the *Vibrio cholerae* zonula occludens toxin [15]. Zonulin is a secretory protein, identical to prehaptoglobin 2, acting as a major regulator of TJ function, and apparently being present in serum and most tissues. For example, when secreted by intestinal cells, into the gut lumen, it will reversibly increase intestinal TJ paracellular transport [15]. The mechanism involves epidermal growth factor receptor (EGFR) activation, and its pathogenetic relevance is illustrated by the finding that gliadin toxicity in celiac disease includes intestinal release of zonulin and EGFR activation. A pathogenetic role for zonulin has been demonstrated in several other autoimmune diseases [15], [26], [27]. Nevertheless, much of the physiological role of zonulin still remain to be established. We revealed zonulin overexpression along the entire small intestine, preceding the onset of disease. Notably, our results correlate well with observations in T1D [2], [15]. T cells are involved in intestinal immunoregulation by exerting either pro-inflammatory or anti-inflammatory activities. Th1 cells enter the CNS during EAE development and then promote the subsequent infiltration and activation of Th17 cells [28]. There is no clear evidence on inflammatory responses in the gut induced by EAE, while recent studies show the influence of gut microbiota on disease development [11], [29], [30]. We observed an increase in proinflammatory Th1 and Th17 cells in LP, PP and MLN as well as in the spleen already at the early phase of EAE. Furthermore, we revealed decreased levels of Tregs in all studied lymphoid tissues, possibly reflecting a conversion of Treg into Th17 cells [23].

IFN-γ and TNF-α have been suggested to trigger changes in IP by reorganizing TJ proteins [15]. We propose that increased levels of IL-17 in combination with IFN-γ and TNF-α released by Th1 and APC may affect the regulation of TJ proteins resulting in zonulin upregulation. Earlier investigation on BBB of EAE mice has shown zonulin reorganization before the onset of clinical disease at sites of inflammatory cell accumulation [31]. Consistent with these results our findings document, for the first time in EAE, zonulin overexpression in both intestinal epithelial and lamina propria cells, suggesting that the regulation of TJ architecture at not only the BBB, but also the intestine will be crucial to restore homeostasis and facilitate disease recovery.

Recognizing the fact that early IFN-γ production may influence the occurrence of the intestinal inflammation, and in order to avoid any complications of result interpretation that might arise from the use of CFA for immunization, we adoptively transferred EAE-pathogenic MOG_35–55_-reactive, IFN-γ producing, Th1 cells. We then found intestinal changes similar to those observed in the actively immunized mice. Interestingly, adoptive transfer of unprimed or OVA-reactive T cells were incapable of increasing IP or zonulin release, highlighting a key role of circulating autoreactive encephalitogenic T cells for our observations of impaired intestinal integrity in EAE.

Previous studies suggested that changes in intestinal barrier function and microbiota play a role in the triggering of the autoimmune disorders. Conversely, our data provide evidence that increased IP in fact can be a consequence of autoimmune reactions.

Thus, our results imply that once the acute autoimmune reactions associated with MS have been initiated, Th1 cells trafficking to the gut release IFN-γ and trigger an intestinal inflammation favouring differentiation of IL-17 producing T cells. Creating such a proinflammatory intestinal milieu may trigger zonulin release, inducing disassembly of TJ and increasing the epithelial permeability. In turn, increased IP seems to be a constant and early feature of the disease process causing abnormal exposure of lymphoid cells to gut microbiota antigen, and thus triggering the multiorgan process leading to a systemic and chronic disease.

Taken together, we report the novel finding of a “leaky gut” syndrome including zonulin upregulation in the EAE animal model for human MS. Recent reports showing that IBD patients are at higher risk for MS, while the course of the IBD disease is not influenced by the MS [8], [9], is further supporting a key role of the gut in the modulation of CNS autoimmunity.

Although the complete link between the TJ and the pathophysiological state of MS is yet to be clarified, a better understanding of the molecular pathways involved in the healing responses to intestinal barrier disruption will offer innovative approaches to help MS patients re-establish their gut barrier function and manage this devastating chronic disease.

## Materials and Methods

### Animals

Female C57BL/6 mice (8–10 weeks old) were obtained from Taconic M & B A/S, Denmark and bred under specific pathogen-free conditions with a controlled environment (20±1°C, 50%±10% relative humidity, 12∶12- hour light dark cycle) and housed in groups in an open cage system with free access to a standard diet and tap water in the department's animal facility. The trials were carried out in strict accordance with the European Community regulations for animal experiments and were approved by the Malmö/Lund Ethical Review Committee for Animal Experiments, Lund District Court (Permit Number: M211-11). All immunizations were performed under isoflurane anesthesia, and all efforts were made to minimize suffering.

### Induction and assessment of EAE

A synthetic peptide from myelin oligodendrocyte glycoprotein (MOG), amino acids 35–55 (MEVGWYRSPFSRVVHLYRNGK-COOH, Schafer-N, Denmark) was used to induce EAE. Mice were immunized by an intradermal injection with 0.1 ml of an emulsion containing 100 µg peptide in complete Freund's adjuvant (CFA) (H37RA, Difco laboratories, USA) together with i.p. injection of 200 ng pertussis toxin (Sigma-Aldrich, Sweden) on days 0 and 2. The progression of disease was followed and the mice were weighed and examined for clinical signs of EAE in a blinded fashion daily. The signs of EAE were scored into eight categories: 0- no signs of clinical disease; 1- weakness in the tail; 2- paralyzed tail; 3- paresis and gait disturbance; 4- paralysis of one limb; 5- paralysis of two limbs; 6- two limbs paralyzed and paresis of a third limb, but the mouse still able to move forward; 7- quadriplegia, no mobility and moribund state; 8- dead. A group of animals were immunized only with CFA followed by i.p. injection of pertussis toxin on days 0 and 2. At the end of the experiments, the animals were anesthetized with a mixture of Ketamin (0.5 mg/g body weight; Ketalar, Parke-Davis, Sweden) and Azaperon (0.4 mg/g body weight; Stresnil, Janssen-Cilag Pharma, Austria) in 0.15 M NaCl. Approximately 0.7 ml blood was taken by heart puncture into tubes containing 1.5 mg EDTA and 20 000 IU aprotinin (Trasylol, Bayer, Germany) and ice-chilled, and tissues to be analyzed were dissected.

### Adoptive transfer of EAE

Twelve days following immunization with MOG_35–55_ peptide or ovalbumin (OVA) (Sigma-Aldrich, Sweden), emulsified in CFA, the animals were sacrificed and the draining lymph nodes including inguinal and axillary lymph nodes were dissected and single cell suspensions prepared by passing through a cell strainer (BD Biosciences, USA). The cells were cultured in round-bottom 96-well culture plates (5×10^5^/well) containing complete DMEM medium and re-stimulated with 50 µg/ml of the antigens in the presence of 20 ng/ml recombinant IL-12 and 10 ng/ml recombinant IL-2 for 3 days. 1×10^7^ viable lymphocytes were then administered intravenously (i.v.) via tail vein into healthy recipient mice. Another group of animals received freshly prepared and un-stimulated lymph node cells from naive animals as the control. Pertussis toxin was injected to all the recipient mice and they were monitored as mentioned above. The mice were investigated for IP changes and then sacrificed at day 14 post-transfer.

### *In vivo* permeability studies

EAE mice at day 7 (EAE7) and day 14 (EAE14) post immunization, animals with adoptively transferred MOG-reactive T cells (EAETransfer), OVA-reactive T cells and un-stimulated lymph node cells, as well as non-immunized mice (control group) were gavaged with a marker molecule solution containing 1.25 mg/g body weight (BW) FITC labelled bovine serum albumin (FITC-BSA) (Sigma, USA, MW 66 KDa) or 10 µg/gr BW sodium fluorescein (Na-F) (Merck Co., Germany, MW 376 Da), in 0.9% NaCl. The mice were anaesthetized and blood samples were collected at either 1 h for Na-F and 2 h for FITC-BSA after gavage. Marker concentrations in blood plasma were measured in 96-microwell plate (Nunc, Denmark) by spectrophotofluorometer (CytoFluo 2300, Millipore Co., USA) using a filter setup for 485 nm excitation (20 nm bandwidth) and 530 nm emission (25 nm bandwidth); and standard concentrations of FITC-BSA and Na-F were used as references.

### Gut morphological studies

The gut samples taken at autopsy were immediately fixed in 4% phosphate buffered formaldehyde for 24 h and then stored in 70% ethanol. Each part of the small intestine; duodenum (the most proximal part of small intestine), jejunum (middle part of small intestine) and ileum (the most distal part of small intestine) were further divided into 3–4 segments until further processing and embedding into paraffin. Each segment was then cut laterally into 5 µm thick sections. 2–3 sections were collected from each segment. All sections were deparaffinised and stained with haematoxylin and eosin (H&E) according to standard procedures. The sections were photographed by using an Olympus PROVIS microscope (objective 20×) equipped with an Olympus DP50 camera (Olympus, Japan). Totally 7–9 sections from each part of the small intestine were analyzed per animal for morphometric parameters (villus length, crypt depth, muscularis and submucosa thickness) by using ImageJ software (Rasband, W.S., ImageJ, U. S. National Institutes of Health, USA. All villi and crypts were analyzed in whole section. Muscularis and submucosal thickness measurements were done under every other crypts. Total 5 animals per group were analysed and all investigations were performed in a blinded fashion.

### Analysis by flow cytometry

At the end of each experiment, the spleen and MLN were dissected and single cell suspensions were obtained. Peyer's patches were excised from the wall of the small intestine and harvested gently in RPMI 1640 medium containing 10% fetal bovine serum (FBS). The cell suspensions were passed through a cell strainer (BD Bioscience) to remove cell debris and washed twice. To isolate immune cells from intestinal lamina propria (LP), the mouse small intestine was isolated, cleaned from fat and connective tissues, opened longitudinally and washed in PBS, cut into 0.5 cm pieces and shaken in 25 ml EDTA solution at 220 r.p.m for 30 min at 37°C (the parts with Peyer's patches were excluded). The supernatant was discarded and the process was repeated twice and the tissues were washed with harvest medium consisting of RPMI 1640, heat inactivated FBS, 100× HGPG (HEPES, L-glutamine, penicillin/streptomycin, and gentamicin) for 5 min before incubation with collagenase solution (RPMI, FBS, 100× HGPG, 0.5 M CaCl_2_, 0.5 M MgCl_2_, 100 U/ml collagenase) for 45 min at 37°C on a shaker.

All cells were then incubated with anti-CD16/CD32 (clone 93) followed by labelled monoclonal antibodies directed to different murine cell surface markers: FITC-conjugated anti-CD3 (145-2C11), PerCP-conjugated anti-CD4 (GK1.5), APC-conjugated anti-CD25 (PC61.5), FITC-conjugated anti-F4/80 (BM8) and FITC-conjucated anti-CD11c (N418) (all purchased from eBioscience). The transcription factor forkhead box P3 (Foxp3) expression in Tregs was analysed using the PE anti-mouse Foxp3 Staining Set (eBioscience). For analysis of intracellular cytokines, cells were fixed with Cytofix/Cytoperm solution and stained with APC-conjugated anti-IFN-γ (XMG1.2), PE-conjugated anti-IL-17A, PE-conjugated anti-IL-6 (MP5-20F3) and PerCP-conjugated anti-TNF-α (MP6-XT22) (eBioscience). A FACSort flow cytometer was used for acquisition of data and analysis was made with CELLQuest software (BD Biosciences, USA) [21].

### Immunohistochemical analysis of zonulin expression in the small intestine

Five mm thick sections from different parts of paraffin-embedded small intestine (duodenum, jejunum and ileum) were deparaffinized by standard protocols. For antigen retrieval, tissue pre-treatment with pepsin (0.05% (w/v) in 2N HCl) was performed. Incubation with primary rabbit anti-zonulin antibody (Invitrogen, USA) was conducted at RT and followed by PBS-washing. HRP-conjugated anti-rabbit antibody (Dako, Denmark) was used as a secondary antibody. Positive immunohistochemical reactions were revealed using the DAB as a chromogen substrate. The slides were scanned with an Aperio ScanScope, and quantitative analysis of positive pixels/mm^2^ was done using Aperio ImageScope software v11.1.2.752 (Aperio, Vista, CA). The staining intensities were expressed as ratios to relevant control groups within the same batch of staining.

### Statistics

Statistical evaluation was performed using StatView software (SAS, USA). *In vivo* permeability data were analyzed using ANOVA with Bonferroni/Dunn testing. Histological and flow cytometry data were evaluated using nonparametric Mann-Whitney test. In all statistical analyses, P≤0.05 was taken as the level of significance.
